# Development of non-dairy creamer analogs/mimics for an alternative of infant formula using egg white, yolk, and soy proteins

**DOI:** 10.5713/ajas.18.0738

**Published:** 2019-01-02

**Authors:** Xi Huang, Eun Joo Lee, Dong U. Ahn

**Affiliations:** 1 College of Food Science & Technology, Huazhong Agricultural University, Egg Processing Technology Local Joint National Engineering Research Center, National R&D Center for Egg Processing, Wuhan, Hubei 430070, China; 2 Department of Food and Nutrition, University of Wisconsin-Stout, Menomonie, WI 54751, USA; 3 Department of Animal Science, Iowa State University, Ames, IA 50011, USA

**Keywords:** Non-dairy Creamer, Egg Yolk and White, Nutrient Content, Shelf-life, Flavor Acceptability

## Abstract

**Objective:**

A study was conducted to develop non-dairy creamer analogs/mimics using egg white, egg yolk, soy protein and their combinations, and their nutrient content, shelf-life and flavor acceptability were compared.

**Methods:**

Spray dried egg white, egg yolk, and soy protein isolate were purchased from manufacturers and used for the formulae.

**Results:**

The protein contents of the non-dairy creamer analogs/mimics were about 8.5% as calculated. The amounts of oleic and linoleic acid content increased as the amount of yolk increased in the formula, but the increases of polyunsaturated fatty acids were <0.5% of total fat. Addition of egg yolk to the formula increased choline and lutein content in the products, but the amounts were <0.4 mg/g for choline and 4 μg/g for lutein. The lutein in the products continued to decrease over the storage time, and only about 15% to 20% of the 0-month amounts were left after 3 months of storage. Although the thiobarbituric acid reactive substances values of the spray-dried non-dairy creamer analogs/mimics increased as storage time increased, the values were still low. Yellowness, darkness, and egg flavor/odor of the non-dairy creamer analogs/mimics increased as the amount of egg yolk in the formula increased. The overall acceptability of the non-dairy creamer analogs/mimics was closely related to the intensity of egg flavor/odor, but storage improved their overall acceptance because most of the off-odor volatiles disappeared during the storage. Water temperature was the most important parameter in dissolving spray-dried non-dairy creamer analogs/mimics, and 55°C to 75°C was the optimal water temperature conditions to dissolve them.

**Conclusion:**

Higher amounts of yolk and soy protein combinations in place of egg white reduced the cost of the products significantly and those products contained better and balanced nutrients than the commercial coffee creamers. However, off-flavor and solubility were two important issues in the products.

## INTRODUCTION

Many poor mothers in the poverty-ridden areas of the world feed their babies and toddlers non-dairy coffee creamers as a substitute for breast milk because most poor mothers cannot produce enough breast milk for their baby due to mom’s malnutrition conditions and the price of infant milk formulas are too expensive for them. However, feeding non-dairy coffee creamers to the poor infants cause severe malnutrition problems because of the low nutritional value of non-dairy creamers. Non-dairy coffee creamers are the substituents for milk or cream as an additive to coffee, tea or hot chocolate. Normally non-dairy coffee creamers contain corn syrup solids (60% to 65%), vegetable oil (30%, hydrogenated coconut or palm oil), sodium caseinate (2% to 5%) and other ingredients (emulsifiers, stabilizers, anti-caking agents, and color and flavoring agents). This means that non-dairy coffee creamers have very low protein content compared with human breast milk (dried), which contain 58% carbohydrate, 32.5% fat, 8.5% protein, and 1.2% minerals. Thus, improvement in nutritional composition in the non-dairy creamers is necessary if they are going to be used as an alternative to the milk formulas.

Egg white is mainly composed of proteins with high levels of limiting amino acids such as methionine, cysteine and lysine. Egg yolk (dried) contains about 61% of lipids, 30% of protein, and 4% of water and 3% of ash [1]. Yolk lipid can be further divided into three main parts, which are neutral lipids (65%), phospholipids (32%), and cholesterol (3%) [2,3]. Phospholipids are the major component of biological membranes and nerve cells, and thus are important nutrients for pre- and postnatal infants whose nerve-system is developing quickly [4]. A diet enriched with egg-derived phospholipids significantly improved the endothelial vasodilatory function [5], reduced arthritis and inflammatory reactions, and had a healing effect on hepatitis A, B, and C [6–8].

Egg yolk is an excellent dietary source of choline, lutein and zeaxanthin. Choline is not an essential nutrient in humans, but dietary deficiency can cause various diseases such as fatty liver, increased lymphocyte apoptosis, and DNA and muscle damages in humans [9,10]. Lutein in egg yolk lipids has been identified as a carotenoid that accumulates in the macular region of the human retina and plays an important role in the prevention of age-related macular degeneration that causes blindness [11–13]. Liu et al [14] reported that lutein and zeaxanthin supplementation improved visual performance of age-related macular degeneration patients in a dose-response manner. One egg yolk contains between 300 and 500 μg of xanthophylls [15], and lutein and zeaxanthin in egg are highly bioavailable because they are dispersed in lipid matrix such as triglycerol, phospholipids, and cholesterol [16]. Egg yolk contains high levels of long-chain omega-3 fatty acids such as eicosapentaenoic acid (EPA) and docosahexaenoic acid (DHA) because of its high phospholipids content. Neuringer et al [17] and Innis [18] reported that infants who do not get enough omega-3 fatty acids from their mothers during pregnancy are at risk for developing vision and nerve problems. Since eggs are an excellent source of high-quality protein and other essential nutrients, the addition of dried egg white and/or yolk can improve the nutritional value of a non-dairy creamer analogs/mimics greatly. When dried egg white and yolk are used together, the improvement of nutritional value will be greater than using egg white alone because egg yolk contains high levels of essential fatty acids, phospholipids, choline, lutein, and minerals, which are important nutrients for infants and growing babies.

Almost half of all deaths in children under 5 worldwide are due to malnutrition and the majority of them are living in Sub-Saharan Africa and South Asia [19]. More than 80% of the worlds’ population lives in poor countries with <$10 a day and almost half of the world population live on a daily income of less than $2.50 [20]. Due to the poverty, malnutrition is one of the biggest social problems in the poverty-ridden areas and many children suffer from one or more forms of malnutrition. The Food and Agriculture Organization indicated that about 154.8 million (22.9%) and 51.7 million children (7.7%) under 5 in the world suffer from stunted growth and wasting, respectively, due to malnutrition [21].

The objectives of this study were i) to develop non-dairy creamer analogs/mimics using dried egg white and/or yolk along with a blend of soy to minimize cost, ii) to determine nutrient (proximate analysis) of the non-dairy creamer analogs/mimics containing eggs and soy proteins, and iii) to measure flavor and sensory acceptability of the non-dairy creamer analogs/mimics produced.

## MATERIALS AND METHODS

### Preparation of non-dairy creamer analogs/mimics formula

Spray dried egg white, egg yolk, and soy protein isolate were purchased from manufacturers and used for the formulae. Modification of formulae and the use of liquid egg white and liquid egg yolk instead of dried ones were also tested. Sodium caseinate level was reduced while the levels of glucose (60%), fat (30%), emulsifier (1.5%), and others (anti-caking agents, gum and flavors, 3.5%) were maintained. The protein content in the formulae was adjusted to approximately 8.5%, and dried egg white, yolk, and soy protein isolate were used as protein sources. When egg yolk was added, smaller amounts of fat (hydrogenated vegetable fat) and emulsifier were used because dried egg yolk contains 61% fat, 1/3 of which is phospholipids, an excellent emulsifier. Soy protein isolate replaced egg white at 1:1 ratio when used.

### Preparation of emulsion and determining emulsion stability

The emulsion was prepared using all the non-dairy formulae (in liquid form) and tested for their stability during storage. The water phase (a mixture of water-soluble compounds), the oil phase, and egg (white and yolk+white) in the non-dairy creamer analogs/mimics formula were prepared separately using Waring blender (22,000 rpm). The water phase was added first, mixed, and then the egg yolk and white were added before adding oil. Emulsion stability was determined by holding the emulsions at 22°C conditions for 7 days after transferring each emulsion to a 100-mL graduated cylinder and observing phase separation over the storage time.

### Spray-drying of non-dairy creamer analogs/mimics

A mini spray dryer (Model B-290, Buchi Labortechnik AG, Postfach, Flawil, Switzerland) was used to spray-dry the non-dairy creamer analogs/mimics. The inlet temperature of the spray dryer was set at 185°C and the exhaust temperature was maintained at 85°C by adjusting the flow rate of liquid non-dairy creamer analogs/mimics to the atomizer. All non-dairy creamer analogs/mimics formulae were freshly prepared, spray dried, subdivided into portions, packaged in oxygen permeable zipper bags, and stored at 22°C for 3 months.

### Chemical analyses

Total lipids and fatty acid composition of the non-dairy creamer analogs/mimics were determined following the method described by Nam et al [22]. Lutein and choline were analyzed using a high performance liquid chromatography method described by Handelman et al [16].

### Volatiles and lipid oxidation

Volatiles of samples were analyzed using the method of Ahn et al [23]. The thiobarbituric acid reactive substances (TBARS) method described by Du and Ahn [24] was used to analyze the extent of lipid oxidation in non-dairy cream powders.

### Sensory evaluation

Ten trained sensory panelists characterized solubility, color intensities including yellowness and darkness, off-odor and off-flavors including beany, egg, and oxidized oil flavors, overall acceptance of the non-dairy creamer analogs/mimics. Sensory panelists were asked to rate the intensity of flavor and color on 9-point scales (1: none of the attribute, 9: extremely high intensity of the attribute). The term of each sensory attribute was provided for panelists. Thirty grams of non-dairy creamer analogs/mimics was dissolved in 300 mL of hot water (70°C), and 20 mL/person was served in a covered 60 mL plastic cup labeled with a random three-digit code. Panelists instructed to smell the samples first and then taste in the randomized order presented on the questionnaire. Participants instructed to rinse their mouths with water before starting to taste, and also between samples. A commercial non-dairy creamer was used as a reference.

### Statistical analysis

Data were analyzed using the SAS software (version 9.2, SAS Institute Inc., Cary, NC, USA). Data were reported as means and standard error of the means. Student-Newman-Keuls’ multiple-range test (p<0.05) was used to compare the means of each treatment.

## RESULTS AND DISCUSSION

### Preparation of non-dairy creamer analogs/mimics

The solubility test of the ingredients used in the non-dairy creamer analogs/mimics formula (Table 1) indicated that some of the ingredients including spray-dried egg yolk, spray-dried egg white, and starch had low solubility and caused serious problems in making emulsions. Significant portions of spray-dried egg yolk and white powders precipitated, and almost all the natural starch added were caked at the bottom of the homogenized solution upon holding due to low solubility. To overcome the solubility problems with the dried egg yolk and white powders, the dried egg white and dried egg yolk were replaced with liquid yolk and white, which were also beneficial in lowering the product cost. To solve the solubility issues with natural starch, hydrolyzed starch and low-molecular starch products such as maltodextrin were used instead. The solubility of hydrolyzed or modified starches was much greater than that of the natural starch, but the solubility could not go higher than 25% and the viscosity of the solutions became very high at the concentration. So, corn syrup was tested as a source of carbohydrates. However, one issue with corn syrup was that the final non-dairy creamer analogs/mimics after spray drying were sticky when corn syrup with dextrose equivalent (DE) value of 30 to 40 was used. So, maltodextrin powder with a DE value of 22 was used in place of corn syrup for the non-dairy creamer analogs/mimics.

The temperatures of the water phase, the oil phase, and egg played an important role in the success or failure of the emulsions. Due to the low heat stability of egg white proteins, especially ovotransferrin [2], maintaining the temperature of water and oil phases at <55°C was important for the successful emulsion preparation. The solubility of soy protein and casein was also low, but their solubility issues were solved by soaking them in warm water (55°C) for 20 min before use. Nasser et al [25] reported that micellar casein loses its rehydration properties during storage, and causes lower solubility and longer rehydration time. The insolubility of proteins develops more rapidly when the protein concentration is high and is primarily due to the aggregation of casein micelles [26].

The emulsion stability of the non-dairy creamer analogs/mimics is important because the liquid product should stay in emulsion during the spray-drying period and the dried products should stay in dispersed conditions when used in coffee or as a milk replacer. The emulsions prepared using all the 10 formulae indicated that emulsion stability increased when egg yolk was included in the formula. Especially, WY1 (9% egg white+2.5% egg yolk), WY2 (8% egg white+5% yolk), and WS1 (8% egg white+2% soy protein) produced the most stable emulsions. However, when egg yolk and/or soy protein was added, the color of the emulsion became dark. When silica was added during the emulsion preparation step, it dramatically reduced emulsion stability. Silica is widely used in food industry as an anti-caking or anti-foaming agent or to control the viscosity of the fluids [27], but it worked negatively to the emulsion stability. So, silica was added to the products after spray drying.

Coconut oil and hydrogenated soybean oil were excellent oil sources for non-dairy creamer analogs/mimics because of their high resistance to oxidative changes during heat processing, spray drying and storage [28]. Corn oil did not have any problems in making emulsions at room temperature conditions, but higher than room temperature conditions were needed when hydrogenated soybean oil or hydrogenated coconut oil were used. A lipid-soluble emulsifier (distilled monoglyceride at 0.5% level, the melting point is 63°C to 68°C) was added to the formula to help emulsification after dissolving it in oils at 65°C before use. Mono- and di-glycerides as well as alginates, carrageenans, gums and gelatin are commonly used as emulsifiers/stabilizers in milk products [29]. Dextrin with DE value of 22 and a water-soluble emulsifier (sodium stearoyl lactylate) at 0.3% level each were included in the formula to improve emulsion stability and emulsification, respectively. Phosphate dibasic was added at 1% level to stabilize casein and other proteins [30]. Milk-type flavor at 0.2% level was used as a flavoring agent. Silica was included in the formula at 0.2% level as an anti-caking agent.

After spray-drying, the non-dairy creamer analogs/mimics should be easily dissolved in water to be used in coffee or as a milk replacer. However, the non-dairy creamer analogs/mimics dispersed slowly due to the proteins from egg yolk, white, and soy proteins even in hot water. The spray-dried non-dairy creamer analogs/mimics behaved like dried milk powders: once they were dissolved in hot water, they remained as suspension/emulsion form even the solution temperature went down to the room temperature. Another important parameter in dissolving non-dairy analogs/mimics was water temperature. If the water temperature was higher than 80°C, some of the protein in the powder started to denature (coagulated). However, it was difficult to dissolve the powder when the water temperature was lower than 45°C because the melting point of coconut oil is 43°C. The best water temperature to make liquid non-dairy creamer analogs/mimics using the dried products was 55°C to 75°C. It was expected that the solubility of the spray-dried non-dairy analog/mimic powders could be improved if a second emulsification/ homogenization step (pressurized or ultrasound homogenizer) is added in the processing line like in the commercial non-dairy creamer producing processes [31].

### Nutrient content, quality and sensory characteristics of non-dairy creamers with storage

The nutrient content of the 10 formulae was within the calculated value ranges with some differences depending upon the formula (protein 8.6% to 10.2%, fat 20.5% to 31.0%, carbohydrate 56.1% to 64.5%, moisture 1.9% to 4.0%, and ash 1.4% to 1.9% ranges, except for the control that has only 3% proteins, Table 2). More than 99% of the fatty acids in the products with no egg yolk in the formula were saturated fatty acids because coconut oil was used in the formula (Table 3). The majority of fatty acids in coconut oil as well as non-dairy creamer analogs/mimics were lauric (C12:0, 41% to 50%), myristic (C14:0, 16% to 18%), palmitic (C16:0, 9% to 12%), stearic (C18:0, 9% to 11%), caprylic (C8:0, 7% to 8%), and capric acid (C10:0, 5% to 6%). As expected, the amounts of oleic (up to 4.8%) and linoleic acid (up to 2.8%) content increased as the amount of egg yolk increased in the formulae, but the increases of polyunsaturated fatty acids were very small (<0.5% of total fat). Preparation of non-dairy creamer analogs/mimics using egg white, egg yolk and egg yolk+soy proteins increased the amount of proteins by 3 folds (from 3% to 9%–10%) and lowered fat content by 6% to 8% from the control. The composition of fatty acids was also changed, but the increases of long-chain omega-3 fatty acids such as EPA and DHA due to the added egg yolk were small (<2%, Table 2).

Addition of 2.5% egg yolk to the formulae increased the choline content in the products by about 10 mg choline/100 g dried products and the amount reached to around 70 mg choline/100 g product when 7.5% of egg yolk was used in the formulae. This amount is 1.5-fold higher than that of the control and >2-fold of the non-dairy creamer analogs/mimics added with egg white. Storage time did not affect the content of choline in the non-dairy creamer analogs/mimics (Table 4). The amounts of lutein in the non-dairy creamer analogs/mimics were very small (<4 μg/g sample) and the amount was the highest when 7.5% egg yolk was added in the formula (Table 4). Although, egg yolk contains about 80 to 120 μg lutein/g yolk depending upon hen’s diet, the non-dairy creamer analogs/mimics added with 7.5% yolk had only 4 μg lutein per gram non-dairy creamer analogs/mimics at 0 month. The amounts of lutein continued to decrease over the storage time, and only about 15% to 20% of the 0-month amounts were left after 3 months of storage. This amount was much smaller than the expected value probably because some of the lutein from the egg yolk should have been destroyed by the heat during the spray-spraying process. Also, more than half of the lutein in spray-dried yolk was destroyed after 3 months of storage. Wang et al [32] and Kuang et al [33] reported that lutein is unstable to heat but encapsulation increase the heat stability of lutein during the spray-drying process as well as storage.

The TBARS values of the spray-dried non-dairy creamer analogs/mimics at 0 month were <1.0 mg/kg product (Table 5). Control had the lowest TBARS value while the non-dairy creamer analogs/mimics with the highest level of soy protein (WS2, 4% soy protein) had the highest TBARS value. The TBARS values of the spray-dried non-dairy creamers analogs/mimics increased from 0.95 to 1.35 in the WS2 formula as the storage time increased from 0-month to 3 months. The TBARS values of the all 9 non-dairy creamer analogs/mimics were significantly higher than that of the control at all storage times. However, the TBARS values were still low and the majority of the general public may not recognize oxidation flavor from the product.

Emulsions are thermodynamically unstable and prone to oxidative changes [34]. The spray-dried non-dairy creamer analogs/mimics prepared from the emulsion are also expected to be more susceptible to oxidative changes because of the increased surface area and air inclusion in the dried powder, which accelerate lipid oxidation [24,35,36]. The inclusion of egg yolk in the formulae of non-dairy creamer analogs/mimics was expected to accelerate lipid oxidation further because egg yolk usually contains phospholipids with high levels of polyunsaturated fatty acids [37]. However, the increase of TBARS in the spray-dried non-dairy creamer analogs/mimics during the storage was relatively small even though they were stored in oxygen-permeable bags at room temperature conditions, which was unexpected. Also, the addition of egg yolk to the formulae of the non-dairy creamer analogs/mimics did not increase the TBARS values, but the addition of soy proteins did (Table 5). The reason for the increase in TBARS of non-dairy creamer analogs/mimics with the soy proteins is not clear, but could be related to the oxidized lipids in the dried soy protein powders.

Volatiles results indicated that ketones were the major volatiles, and aldehydes and hydrocarbons were the next highest volatiles produced from the non-dairy creamer analogs/mimics at 0 month. Only very small amounts of alcohols and sulfur compounds were detected at 0 month (Table 6). The amounts of ketones were not influenced by non-dairy creamer analogs/mimics formulae and had little effect on the odor/flavor of non-dairy creamer analogs/mimics. Six different aldehydes, which include propanal, butanal, 3-methyl butanal, 2-methyl butanal, pentanal and hexanal, were produced from the non-dairy creamers analogs/mimics. The amounts of butanal, 3- methyl butanal, 2-methyl butanal and hexanal increased as the amount of egg yolk and soy protein in formulae increased. Aldehydes, especially hexanal, are usually closely related to the degree of lipid oxidation in food products [38,39]. No consistent trends for the amounts and kinds of hydrocarbons by the non-dairy creamer analogs/mimics formulae were observed. Carbon disulfide was the only sulfur compound detected from the non-dairy creamer analogs/mimics at low levels at 0-month storage (Table 6). Ahn [40] reported that sulfur compounds are responsible for the typical “hard-boiled egg” smell. Sulfur compounds easily disappear during storage in oxygen permeable packaging conditions because they are highly volatile [41]. The profiles and amounts of volatiles in the non-dairy creamer analogs/mimics after 1-month storage were not much different from those of the 0-month samples, except for the dramatic increase in hydrocarbons in soy protein-added formulae (data not shown). The amounts of total and individual volatile, and volatile profiles of the all 10 non-dairy creamer analogs/mimics changed significantly after 3-month storage. The amounts of volatiles in non-dairy creamer analogs/mimics after 3 months of storage were less than a half of the 0-month samples and many hydrocarbons and all the sulfur compounds disappeared (Table 6).

The sensory results of the non-dairy creamer analogs/mimics at 0-month storage are as follow: yellowness and darkness of the liquid non-dairy creamer analogs/mimics increased as the amount of egg yolk in the formula increased (Table 7). Also, sensory panels sensed egg flavor/odor in all egg white and/or yolk added products. The intensity of egg flavor/odor in the formula with egg white alone was very weak. However, the intensity increased as the amount of egg yolk in the formula increased. With 7.5% of egg yolk in the formula, the intensity was rated as ‘strong’ egg flavor/odor. No beany flavor/odor or oxidized oil flavor/odor was detected, or negligible if any. The overall acceptability of the non-dairy creamer analogs/mimics was ‘high’ to ‘very high’ except for 7.5% egg yolk-added formulae (somewhat acceptable). The overall acceptability of the non-dairy creamer analogs/mimics was closely related to the intensity of egg flavor/odor (Table 7). Pasteurization of the emulsion before spray-drying, which is a mandatory step for the commercial processing, may reduce the egg flavor/odor problems in the non-dairy creamer analog/mimic products as well. Some of the formulae containing high levels of egg yolk produced egg flavor/odor if not stored for 3 months in oxygen-permeable bags. Oxidation of the fats in the powder was minimal probably because coconut oil was used as the main fat source, but long-term storage in oxygen-permeable bags can cause flavor problems if other fat sources with high polyunsaturated fatty acids are used.

Because the solubility of the non-dairy creamer analogs/mimics, except for the control (commercial), were not different from each other and the color of the non-dairy creamer analogs/mimics did not change over the storage time, solubility and color of the products were not measured after 0-month storage. Beany flavor/odor was detected from the non-dairy creamer analogs/mimics at 0 month of storage, but disappeared after 3 months of storage probably due to the decrease in aldehydes during the storage (Table 6). Ketones were the major volatiles at 0-month storage, but they had no effect on the odor of the non-dairy creamer analogs/mimics because of they have high thresholds [42]. Takahashi et al [43] reported that aldehydes (hexanal and pentanal) are responsible for the beany flavor. Oxidized flavor/odor increased slightly during the storage, but the intensity was very weak in all the formulae. Egg flavor/odor in egg yolk-added formulae was a concern at 0 month, but became non-significant after 3 months of storage. Due to a significant decrease in the egg flavor/odor in egg yolk-added formulae in aerobic storage conditions, which lost all the sulfur compounds that are responsible for the sulfury hard-boiled egg odor [40], overall acceptance of all the non-dairy creamer analogs/mimics were greater than 5 (moderately high). This indicated that storage improved the overall acceptance of non-dairy creamer analogs/mimics because most of the off-odor volatiles disappeared during the storage.

One of the underlying objectives of this study was developing non-dairy creamer analogs/mimics using egg products to improve the nutrient quality of the non-dairy creamer analogs/mimics and use it as a low-cost milk replacer for the low-income family. If the non-dairy creamer analogs/mimics developed are going to be used as a milk replacer, however, the balance of nutrients and their composition (polyunsaturated fatty acids such as EPA and DHA, choline, calcium, lutein, immunoglobulin, etc.) should be further improved by adding more egg yolk. Also, methods that could increase the solubility and minimize or eliminate egg flavor from the products should be developed. The cost analysis for the formulae indicated that the more liquid egg white was used, the higher the cost of the product would be. However, egg yolk was a cost-relieving ingredient. Higher amounts of yolk and soy protein combinations in place of egg white reduced the cost of the products close to the level of commercial non-dairy coffee creamers analogs/mimics.

## CONCLUSION

It is possible to make acceptable non-dairy creamer analogs/mimics using egg yolk, egg white, soy protein, or their combinations. The non-dairy creamer analogs/mimics produced in this study can not only be used as a coffee creamer but also be used as a substitute for breast milk with superior nutritional quality to the commercial coffee creamer analogs/mimics. Addition of more egg yolk components to the formula can improve the nutritional quality and reduce the production costs of the non-dairy creamer analogs/mimics, but increase in egg flavor/odor and decrease in solubility can be important issues to overcome. Solving the solubility, off-flavor and nutrient balances, especially in fatty acid composition, are important issues to overcome if the non-dairy creamer analogs/mimics are going to be used as a coffee creamer as well as a milk replacer.

## Figures and Tables

**Table 1:** The formula of non-dairy creamers

Ingredient	C1)	W1)	WY11)	WY21)	WY31)	WS11)	WS21)	WYS11)	WYS21)	WYS31)

	------------------------------------------------------------------ % in formula -------------------------------------------------------------------									
Glucose	65	57	57	57	57	57	57	57	57	57
Oil	30.5	29	28	27	26	29	29	28	27	26
Egg yolk	0	0	2.5	5	7.5	0	0	2.5	5	7.5
Egg white	0	10	9	8	7	8	6	7	6	5
Soy protein	0	0	0	0	0	2	4	2	2	2
Sodium caseinate	2	1.5	1	0.5	0	1.5	1.5	1	0.5	0
Phosphate, dibasic	1	1	1	1	1	1	1	1	1	1
MG	0.5	0.5	0.5	0.5	0.5	0.5	0.5	0.5	0.5	0.5
Dextrin	0.3	0.3	0.3	0.3	0.3	0.3	0.3	0.3	0.3	0.3
SSL	0.3	0.3	0.3	0.3	0.3	0.3	0.3	0.3	0.3	0.3
Silica	0.2	0.2	0.2	0.2	0.2	0.2	0.2	0.2	0.2	0.2
Flavoring agent	0.2	0.2	0.2	0.2	0.2	0.2	0.2	0.2	0.2	0.2
Total	100	100	100	100	100	100	100	100	100	100

MG, monoglyceride; SSL, sodium stearoyl lactylate.

1) C, control; W, 10% egg white; WY1, 9% egg white+2.5% egg yolk; WY2, 8% egg white+5% yolk; WY3, 7% egg white+7.5% egg yolk; WS1, 8% egg white+2% soy protein; WS2, 6% egg white+4% soy protein; WYS1, 7% egg white+2.5% egg yolk+2% soy protein; WYS2, 6% egg white+5% egg yolk+2% soy protein; WYS1, 5% egg white+7.5% egg yolk+2% soy protein.

**Table 2:** The nutrient compositions of non-dairy creamers with different formula

Nutrient	C1)	W1)	WY11)	WY21)	WY31)	WS11)	WS21)	WYS11)	WYS21)	WYS31)	SEM
Moisture (%)	3.2^b^	4.0^a^	2.3^bc^	3.1^bc^	2.3^bc^	2.1^c^	1.9^c^	2.3^bc^	2.2^bc^	2.7^bc^	0.3
Ash (%)	1.4^c^	1.9^a^	1.7^b^	1.7^b^	1.8^a^	1.8^a^	1.9^a^	1.8^a^	1.7^b^	1.5^bc^	0.1
Fat (%)	31.0^a^	20.5^e^	22.1^d^	30.0^ab^	30.7^ab^	22.4^d^	23.8^d^	27.1^c^	28.8^b^	29.2^ab^	0.5
Protein (%)	3.0^e^	10.2^a^	9.4^b^	9.1^c^	8.6^d^	10.2^a^	10.1^a^	9.4^b^	9.2b^c^	8.5^d^	0.1
Carbohydrate (%)	61.3^c^	63.4^ab^	64.5^a^	56.1^e^	56.5^e^	63.5^ab^	62.2^bc^	59.4^d^	58.0^de^	58.1^de^	0.5
Total (%)	100	100	100	100	100	100	100	100	100	100	-

SEM: standard error of the mean. n = 4.

1) C, control; W, 10% egg white; WY1, 9% egg white+2.5% egg yolk; WY2, 8% egg white+5% yolk; WY3, 7% egg white+7.5% egg yolk; WS1, 8% egg white+2% soy protein; WS2, 6% egg white+4% soy protein; WYS1, 7% egg white+2.5% egg yolk+2% soy protein; WYS2, 6% egg white+5% egg yolk+2% soy protein; WYS1, 5% egg white+7.5% egg yolk+2% soy protein.

a–e Values with different superscripts within a row are significantly different (p<0.05).

**Table 3:** The fatty acid composition of non-dairy creamers with different formula

Fatty acid	C1)	W1)	WY11)	WY21)	WY31)	WS11)	WS21)	WYS11)	WYS21)	WYS31)	SEM
	-------------------------------------------------------------- % of total fat ------------------------------------------------------------------										
Caprylic acid (C8:0)	8.0^a^	7.9^a^	7.2^bc^	7.2^bc^	6.8^cd^	7.6^ab^	7.6^ab^	7.6^ab^	7.1^bc^	6.5^d^	0.1
Capric acid (C10:0)	6.5^a^	6.4^ab^	5.9^cd^	5.8cde	5.4^e^^f^	6.2abc	6.2abc	6.0bcd	5.6^de^	5.2^f^	0.1
Lauric acid (C12:0)	49.7^a^	49.4^a^	46.4^bc^	45.2^cd^	42.4^e^	48.4^ab^	48.3^ab^	46.6^bc^	44.2^d^	41.0^e^	0.6
Myristic acid (C14:0)	17.8^a^	17.9^a^	17.7^a^	17.0^b^	16.2^c^	18.2^a^	18.2^a^	17.7^a^	17.0^b^	16.0^c^	0.1
Palmitic acid (C16:0)	8.7^e^	8.9^e^	10.3bcd	10.7^bc^	11.8^a^	9.4^de^	9.5^de^	10.0^cd^	11.1^b^	12.2^a^	0.2
Stearic acid (C18:0)	8.5^bc^	8.8^bc^	10.1^ab^	9.8abc	10.2^ab^	9.6abc	9.5abc	9.8abc	10.3^ab^	10.8^a^	0.4
Oleic acid (C18:1)	0.3^e^	0.3^e^	1.5^d^	2.5^c^	4.2^b^	0.4^e^	0.6^e^	1.4^d^	2.7^c^	4.8^a^	0.2
Linoleic acid (C18:2)	0.0^e^	0.0^e^	0.8^d^	1.5^c^	2.5^b^	0.0^e^	0.0^e^	0.7^d^	1.6^c^	2.8^a^	0.1
Linolenic acid (C18:3)	0	0.1	0	0.1	0.1	0	0	0	0.1	0.1	0
Arachidic acid (C20:0)	0.4	0.4	0.1	0.1	0.2	0.2	0.1	0.1	0.1	0.2	0.1
Arachidonic acid (C20:4)	0.0^c^	0.0^c^	0.1^b^	0.1^b^	0.2^a^	0.0^c^	0.0^c^	0.0^c^	0.1^b^	0.2^a^	0

SEM, standard error of the mean. n = 4.

1) C, control; W, 10% egg white; WY1, 9% egg white+2.5% egg yolk; WY2, 8% egg white+5% yolk; WY3, 7% egg white+7.5% egg yolk; WS1, 8% egg white+2% soy protein; WS2, 6% egg white+4% soy protein; WYS1, 7% egg white+2.5% egg yolk+2% soy protein; WYS2: 6% egg white+5% egg yolk+2% soy protein, WYS1: 5% egg white+7.5% egg yolk+2% soy protein.

a–e Values with different superscripts within a row are significantly different (p<0.05).

**Table 4:** Choline and lutein contents of non-dairy creamers with different formula and storage time

Items	C1)	W1)	WY11)	WY21)	WY31)	WS11)	WS21)	WYS11)	WYS21)	WYS31)	SEM
Choline (month)	--------------------------------------------------------- mg choline hydroxide/100 g sample -------------------------------------------------------										
0	45.57^d^	32.61^f^	40.36^e^	51.45^bc^	61.07^a^	35.61^ef^	35.43^ef^	47.92^cd^	54.79^b^	59.94^a^	1.63
1	39.56^e^	33.28^g^	46.86^d^	59.77^c^	79.36^a^	36.08^f^	35.11f^g^	46.79^d^	63.70^b^	78.89^a^	0.76
2	42.98^e^	32.93^f^	44.74^de^	57.11^c^	73.42^a^	35.65^f^	34.73^f^	46.34^d^	60.66^b^	73.63^a^	0.85
3	41.27^e^	32.58^f^	42.62^e^	54.45^c^	67.49^a^	35.22^f^	34.35^f^	45.89^d^	57.63^b^	68.37^a^	0.95
Lutein (month)	---------------------------------------------------------------------- μg/g sample --------------------------------------------------------------------										
0	0.00^e^	0.00^e^	0.57^d^	1.76^c^	3.82^a^	0.00^e^	0.00^e^	0.46^d^	1.87^c^	3.31^b^	0.07
1	0.00^d^	0.00^d^	0.27^d^	1.28^c^	2.43^a^	0.00^d^	0.00^d^	0.12^d^	1.03^c^	1.96^b^	0.1
2	0.00^g^	0.00^g^	0.21^e^	0.79^c^	1.29^a^	0.00^g^	0.00^g^	0.10^f^	0.52^d^	1.00^b^	0.01
3	0.00^e^	0.00^e^	0.01^e^	0.34^c^	0.66^a^	0.00^e^	0.00^e^	0.02^e^	0.28^d^	0.62^b^	0.01

SEM, standard error of the mean. n = 4.

1) C, control; W, 10% egg white; WY1, 9% egg white+2.5% egg yolk; WY2, 8% egg white+5% yolk; WY3, 7% egg white+7.5% egg yolk; WS1, 8% egg white+2% soy protein; WS2, 6% egg white+4% soy protein; WYS1, 7% egg white+2.5% egg yolk+2% soy protein; WYS2, 6% egg white+5% egg yolk+2% soy protein; WYS1, 5% egg white+7.5% egg yolk+2% soy protein.

a–g Values with different superscripts within a row are significantly different (p<0.05).

**Table 5:** The TBARS values of non-dairy creamers with different formula and storage time

Storage (month)	C1)	W1)	WY11)	WY21)	WY31)	WS11)	WS21)	WYS11)	WYS21)	WYS31)	SEM
	---------------------------------------------------------------- mg MDA/kg sample -----------------------------------------------------------------										
0	0.40^d^	0.72^bc^	0.55^c^	0.66^c^	0.66^c^	0.68^bc^	0.95^a^	0.70^bc^	0.84^ab^	0.85^ab^	0.04
1	0.64^d^	1.00^ab^	0.88^bc^	0.76^cd^	0.98abc	0.81bcd	1.11^a^	0.86^bc^	0.98abc	1.12^a^	0.10
2	0.55^e^	1.01^c^	0.95^c^	0.80^d^	0.95^c^	0.92^c^	1.23^a^	0.96^c^	1.11^b^	1.19^ab^	0.03
3	0.46^d^	1.01^b^	1.03^b^	0.84^c^	0.93^bc^	1.02^b^	1.35^a^	1.06^b^	1.23^a^	1.27^a^	0.04

SEM, standard error of the mean. n = 4.

1) C, control; W, 10% egg white; WY1, 9% egg white+2.5% egg yolk; WY2, 8% egg white+5% yolk; WY3, 7% egg white+7.5% egg yolk; WS1, 8% egg white+2% soy protein; WS2, 6% egg white+4% soy protein; WYS1, 7% egg white+2.5% egg yolk+2% soy protein; WYS2, 6% egg white+5% egg yolk+2% soy protein; WYS1, 5% egg white+7.5% egg yolk+2% soy protein.

a–d Values with different superscripts within a row are significantly different (p<0.05).

**Table 6:** The volatile compounds of non-dairy creamers with different formula and storage time

Items	C1)	W41)	WY11)	WY21)	WY31)	WS11)	WS21)	WYS11)	WYS21)	WYS31)	SEM
0-month storage	---------------------------------------------------------- Total ion counts ×10^4^ -------------------------------------------------------------										
Aldehydes	1,813^f^	3,523^e^	3,673^e^	6,131^d^	1,0533^b^	4,139^e^	7,598^c^	8,202^c^	1,2148^a^	6,520^d^	226
Alcohols	0^d^	0^d^	332^bc^	342^b^	402^a^	298^bc^	280^c^	345^b^	0^d^	0^d^	15
Ketones	15,742^b^	15,461^b^	14,361^b^	14,829^b^	14,058^b^	15,056^b^	15,308^b^	18,114^a^	13,379^b^	10,063^c^	590
Hydrocarbons	323^b^	179^d^	419^a^	363^ab^	390^a^	413^a^	368^ab^	248^c^	167^d^	172^d^	17
Sulfur compounds	151^a^	124^b^	0^d^	108^bc^	99^c^	106^bc^	110^bc^	95^c^	108^bc^	0^d^	6
Others	1,668^b^	1,920^a^	1,106^e^	783^f^	1,296^d^	1,442^cd^	1,536^c^	1,344^d^	1,442^cd^	1,077^e^	45
Total volatiles	19,697^de^	21,206^cd^	19,891^de^	22,556^c^	26,778^ab^	21,454^cd^	25,200^b^	28,349^a^	27,243^ab^	17,832^e^	665
3-month storage											
Aldehydes	151^f^	676^e^	2,036^c^	2,441^b^	3,685^a^	969^e^	0^f^	1,578^d^	3,390^a^	3,407^a^	126
Alcohols	1,238^c^	2,125^bc^	2,021^bc^	1,687^bc^	1,956^bc^	3,759^a^	3,265^a^	3,325^a^	2,175^bc^	2,730^ab^	281
Ketones	1,574^a^	1,520^a^	1,340^a^	2,006^a^	1,842^a^	1,744^a^	577^b^	1,535^a^	1,864^a^	2,000^a^	157
Hydrocarbons	118^de^	138cde	241^a^	91^e^	244^a^	0^f^	0^f^	179^bc^	211^ab^	159bcd	15
Others	1,802^d^	2,407^d^	2,233^d^	2,507^d^	2,386^d^	1,939^d^	6,839^c^	8,421^b^	9,485^a^	2,608^d^	304
Total volatiles	4,883^f^	6,866^e^	7,959^de^	8,732cde	10,191^cd^	8,411cde	10,681^c^	15,038^b^	17,124^a^	10,903^c^	612

SEM, standard error of the mean. n = 4.

1) C, control; W, 10% egg white; WY1, 9% egg white+2.5% egg yolk; WY2, 8% egg white+5% yolk; WY3, 7% egg white+7.5% egg yolk; WS1, 8% egg white+2% soy protein; WS2, 6% egg white+4% soy protein; WYS1, 7% egg white+2.5% egg yolk+2% soy protein; WYS2, 6% egg white+5% egg yolk+2% soy protein; WYS1, 5% egg white+7.5% egg yolk+2% soy protein.

a–f Values with different superscripts within a row are significantly different (p<0.05).

**Table 7:** The sensory evaluation1) of non-dairy creamers with different formula and storage

Items	C2)	W2)	WY12)	WY22)	WY32)	WS12)	WS22)	WYS12)	WYS22)	WYS32)	SEM
0-month storage											
Solubility	6^a^	5^b^	4^cd^	5^b^	5^b^	5^b^	5^b^	4^bc^	3^d^	3^d^	0.3
Color											
Yellowness	2^c^	2^c^	4^b^	5^ab^	6^a^	2^c^	3^c^	5^b^	5^b^	6^a^	0.3
Darkness	2^d^	3^cd^	4^bc^	4^bc^	6^a^	3bcd	4^bc^	4^b^	4^b^	5^a^	0.3
Off-odor/flavor											
Beany flavor/odor	1	2	2	2	2	1	2	2	2	2	0.3
Egg flavor/odor	1^c^	2^c^	3^b^	3^b^	6^a^	1^c^	2^c^	3^b^	3^b^	6^a^	0.3
Oxidized oil flavor/odor	1	1	1	2	2	1	1	1	1	2	0.3
Overall acceptance	6^ab^	6^ab^	6^ab^	5^bc^	4^c^	6^ab^	6^a^	5^ab^	5^bc^	4^c^	0.4
33-month storage											
Off-odor/flavor											
Egg flavor/odor	1^c^	2^bc^	2^bc^	4^a^	3^ab^	2^bc^	2^bc^	2^bc^	2^bc^	4^a^	0.3
Oxidized oil flavor/odor	1	2	1	2	2	1	2	2	2	2	0.3
Overall acceptance	7^a^	6^b^	5^b^	5^b^	6^b^	6^b^	5^b^	5^b^	5^b^	5^b^	0.3

SEM, standard error of the mean. n = 10.

1) Sensory scores: 1, none; 3, slightly; 5, moderately; 7, very much; 9, extremely.

2) C, control; W, 10% egg white; WY1: 9% egg white+2.5% egg yolk; WY2, 8% egg white+5% yolk; WY3, 7% egg white+7.5% egg yolk; WS1, 8% egg white+2% soy protein; WS2, 6% egg white+4% soy protein; WYS1, 7% egg white+2.5% egg yolk+2% soy protein; WYS2, 6% egg white+5% egg yolk+2% soy protein; WYS1, 5% egg white+7.5% egg yolk+2% soy protein.

a–d Values with different superscripts within a row are significantly different (p<0.05).
